# Early detection of Alzheimer’s disease using neuropsychological tests: a predict–diagnose approach using neural networks

**DOI:** 10.1186/s40708-022-00169-1

**Published:** 2022-09-27

**Authors:** Devarshi Mukherji, Manibrata Mukherji, Nivedita Mukherji, Alzheimer’s Disease Neuroimaging Initiative

**Affiliations:** 1 University of Michigan, Ann Arbor, MI USA; 2 Rochester, MI USA; 3 Oakland University, Rochester, MI USA

**Keywords:** Alzheimer’s disease, Recurrent neural networks, Multi-layer perceptron neural networks, Sequence prediction

## Abstract

Alzheimer’s disease (AD) is a slowly progressing disease for which there is no known therapeutic cure at present. Ongoing research around the world is actively engaged in the quest for identifying markers that can help predict the future cognitive state of individuals so that measures can be taken to prevent the onset or arrest the progression of the disease. Researchers are interested in both biological and neuropsychological markers that can serve as good predictors of the future cognitive state of individuals. The goal of this study is to identify non-invasive, inexpensive markers and develop neural network models that learn the relationship between those markers and the future cognitive state. To that end, we use the renowned Alzheimer’s Disease Neuroimaging Initiative (ADNI) data for a handful of neuropsychological tests to train Recurrent Neural Network (RNN) models to predict future neuropsychological test results and Multi-Level Perceptron (MLP) models to diagnose the future cognitive states of trial participants based on those predicted results. The results demonstrate that the predicted cognitive states match the actual cognitive states of ADNI test subjects with a high level of accuracy. Therefore, this novel two-step technique can serve as an effective tool for the prediction of Alzheimer’s disease progression. The reliance of the results on inexpensive, non-invasive tests implies that this technique can be used in countries around the world including those with limited financial resources.

## Introduction

Alzheimer’s disease (AD) is a neurodegenerative disease that affects around 50 million people globally [1]. Projections show that 1 in 85 people will develop AD by 2050. In spite of the scale of the problem, there is no cure for the disease and countless clinical trials have been unsuccessful in finding effective treatment [2, 3]. In 2018, the Alzheimer’s Association estimated that the average cost of caring for an Alzheimer’s disease patient is $350,174—making it the most expensive disease in the United States.

Research shows that AD is an incredibly slowly progressing disease [4–6] and takes many years from initial cognitive decline to full-blown disease development. Due to this slow progression, early detection of AD can be crucial in both its treatment and prevention. Currently, the detection of AD relies on invasive and expensive tests—spinal taps for CSF Tau protein, brain scans, and blood biomarker detections [7–9]. These tests contribute to the overall costs associated with AD assessment and treatment mentioned above. It is therefore critical to develop an effective, less expensive, and nonintrusive screening method to identify people who are at risk of developing AD so that the expensive and intrusive tests can be used only for those patients. Moreover, multiple ongoing global efforts aim to identify the optimal candidates for their clinical trials (cohorts) to test new therapies and understand disease progression. The cohorts selected for these trials often include patients who do not develop cognitive impairment during their participation and result in an unfortunate waste of resources [8, 10].

It has been shown that neuropsychological tests are effective in the diagnosis of AD and in the identification of patients that are likely to experience AD progression [11–17]. These tests are much cheaper to administer than CSF spinal taps and brain scans and can be used to identify patients at risk of developing cognitive decline. The goal of this study is to use multiple Long Short-Term Memory Recurrent Neural Networks (LSTM RNNs, for their ability to handle the vanishing gradients problem and capture long-term dependencies efficiently) on a set of well-established neuropsychological tests such as the ones used by [18] to assess the current cognitive state of a subject and use current and past test score data to predict if a subject is likely to develop cognitive impairment within the next 2 to 4 years. This segmentation of subjects into those who are expected to remain cognitively normal and those likely to develop problems allows doctors and researchers to select appropriate subjects for clinical trials. Access to inexpensive assessments of cognitive state can be of great benefit to people around the world, especially those in underdeveloped countries where low-cost diagnosis methods can be used for screening and more expensive tests can be preserved for only the most high-risk patients.

All data for this study were obtained from the Alzheimer’s Disease Neuroimaging Initiative (ADNI) database. ADNI was launched in 2003 in cooperation with the National Institutes of Health (NIH) and the National Institute on Aging to better understand AD development and discover new therapeutic techniques. This database includes data on neuropsychological tests, along with many other metrics used to monitor AD progression. Based on the research on neuropsychological tests in AD detection and prediction, five different neuropsychological tests (MMSE, ADAS Q4, ADAS Cog11, ADAS Cog13, and FAQ) were chosen for this study—[12] offers a brief description of these tests. The ADNI database includes data on all of these tests. Another study [19], assessed different neuropsychological tests to determine which have good prediction power.

Neural networks have been used in the medical field for several years, and recently data from the ADNI database have been used by many researchers to predict neuropsychological test scores or cognitive status primarily based on brain scans and volumes of different parts of the brain that have been identified as correlated to AD progression [20–30]. Even though our work differs from this body of research in its goal to utilize inexpensive markers like neuropsychological test scores, it does strengthen our hypothesis that ADNI’s data on neuropsychological tests capture the subtle influences of important biomarkers like brain volume and can be used on their own to predict cognitive status.

An interesting research work that reinforces the relative contribution of the ADNI neuropsychological tests on the cognitive status of the trial participants is [31]. The authors use the Fuzzy-Rough Feature Selection technique to determine the most effective set of features that makes the largest contribution towards the cognitive status outcomes arrived at by ADNI. They use two search strategies and three similarity functions to determine the set of features that contribute the most in determining the ADNI outcome. They use a forward search in which features are inserted one at a time and a backward search in which features are removed iteratively. The best forward search feature set includes seven neuropsychological tests of which three are used in our work. The best backward search feature set contains no neuropsychological tests. Moreover, they use eight neural network models (no RNNs) to find the accuracy of these features in modeling the ADNI outcomes. The best forward-pass feature set yields the highest average accuracy of around 85% (Table 4 in the paper) and the best backward-pass feature set yields the lowest average accuracy of around 39% (Table 5 in the paper). The 85 percent accuracy number indicates that the neuropsychological tests contribute disproportionately to the accuracy of the ADNI cognitive status determinations. The 39 percent accuracy number indicates that the absence of neuropsychological tests has a disproportionately adverse effect on the determination of the ADNI cognitive status. Both outcomes corroborate the suitability of using neuropsychological tests on their own in any AD modeling work and reinforce the motivation behind our work.

Other research that uses past test data to predict future patient outcomes includes [32] where a five-step procedure is developed to record the impact of an auditory task on the EEG recordings of schizophrenia patients. The paper attempts to automate the decision-making process of whether to continue therapy for these patients in the future or to stop it. Two databases are used to store therapy outputs over multiple time steps and correlations between decisions made for those time steps. In contrast, our work captures the relationship between the input markers and the normal/abnormal status in the trained neural network models and we do not need any explicit equation to model the relationship between inputs and the predicted future state. Also, there is no storage of any data in a database for future lookup.

As mentioned before, this study uses LSTM RNNs (LSTMs, henceforth) for prediction and Multi-Layer Perceptrons (MLPs) for diagnosis. MLPs are a class of Artificial Neural Networks (ANNs) that use multiple layers of neurons and can be used to classify input data. LSTMs are machine learning models that use sequences of data to predict future values [33–36]. LSTMs have been used effectively in many domains ranging from language recognition to marketing to healthcare. In the context of AD, [37] uses sequence prediction models in which irregularity in time series data are handled by a neural network layer that models a continuous-time autoregressive (CAR) model followed by either an RNN, LSTM, or GRU layer to predict future test values for several markers from the ADNI database. Since our data have no time irregularities, we used just an LSTM to predict the sequence values, and, unlike [37], used the predicted values to perform diagnosis using MLPs. [18] is one of the recent studies that used LSTM in AD prediction. Using National Alzheimer’s Coordinating Center (NACC) data, [18] used 78 features and a global CDR (Clinical Dementia Rating) score to identify patients that are likely to experience AD progression based on changes in CDR scores. Unlike ADNI, the NACC data are not collected at equal intervals for all subjects necessitating [18] to introduce time between visits as a separate feature. The paper utilized a large number of features (78) in a multi-feature model to identify patients that are likely to experience worsening of the disease in the future. In contrast, the LSTMs used in the current study are developed individually for each of the chosen tests, and their output is analyzed using MLPs to diagnose a patient as likely to remain cognitively normal or not in the next 2 to 4 years. Consequently, this paper can be viewed as a less expensive step that should be conducted before a full-blown LSTM that uses a comprehensive and expensive set of features is used to determine how rapidly a patient’s cognitive decline is expected to progress in the future. The objectives of this study are:

1. To develop LSTMs, one for each of the chosen neuropsychological tests in the ADNI dataset, that can predict future test scores based on past values.
2. To develop MLPs that use all predicted test scores from step 1 above to diagnose whether the patient has cognitive impairment.
3. To combine the above two sets of neural network models to predict test scores for each test over the next 2–4 years and diagnose upon those predictions.

All of these objectives were met in this study. In the process of meeting the objectives, we have demonstrated that neuropsychological tests can be used on their own to predict the cognitive state of individuals. The goal of this work was not to supersede the cognitive assessments made by ADNI. Instead, the goal was to determine whether the cognitive assessments made by ADNI could be used as outputs to train MLPs which relied only on a subset of the set of features that ADNI gathers for each participant (namely, neuropsychological tests). Data for these features can be easily gathered and the two-step neural network algorithm can be used to forecast cognitive status for a 2- to 4-year window. In addition, our innovative approach of using predicted test results for diagnosing can be utilized for any set of markers from other established trials going on around the world. There are other studies that have predicted multiple feature values using neural networks, but we think that this work is the first one that used such predicted values as collective inputs to MLPs to diagnose cognitive status.

## Methods

### Data

#### Data, software, and packages

All data used in this project were obtained from the ADNI database. ADNI is a longitudinal study launched in 2003 in cooperation with the National Institutes of Health (NIH) and the National Institute on Aging. ADNI uses adult volunteers between the ages of 55 and 90. The initial cohort included 200 cognitively normal (CN) subjects, 400 subjects with mild cognitive impairment (MCI), and 200 subjects diagnosed with Alzheimer’s disease (AD). Each individual is assigned a unique identification number or RID. CN subjects have a Clinical Dementia Rating (CDR) of 0 and MMSE score between 24 and 30; MCI subjects show signs of memory loss, have a CDR score of 0.5, and are at least one standard deviation below the mean score on the delayed recall portion of the Wechsler Memory Scale’s Logical Memory II. Those who are diagnosed with AD are diagnosed in accordance with the National Institute of Neurological and Communicative Disorders and Stroke-Alzheimer’s Disease and Related Disorders Association (NINCDS-ADRDA) [38]. The ADNI study has completed three phases to date and is currently in its ADNI3 phase. The first phase, ADNI1, started in October 2004 and spanned 5 years. ADNI-GO was the second phase and lasted 2 years after its launch in September 2009, and ADNI2 spanned 5 years since its launch in September 2011. In each phase, new participants were recruited, and participants from the previous phases continued. The ADNI dataset contains demographic data of the subjects as well as results of various neuropsychological tests, results of brain scans, and other physiological tests conducted at various intervals. The relevant data for this study are obtained from a subset of the ADNI dataset called ADNIMERGE which was downloaded on May 14, 2019. Data extraction and processing were conducted using the R programming language running in RStudio version 1.2. The recurrent neural network analysis was conducted using the Keras package version 2.2.5.0 running in RStudio. Descriptive statistical analysis was conducted using the statistical software package Stata 14.

Since the objectives of this paper are based on LSTMs that use past neuropsychological test scores to predict future test scores, it is important to select subjects who have sufficient time series data for the chosen tests. ADNI subjects undergo an initial assessment that determines their baseline cognitive states and then have follow-up tests at months 6, 12, 18, 24, 36, 48, 60, 72, 84, and so on. The data for this study are primarily derived from the baseline, months 6, 12, 24, 36, 48, 60, and 72. (Months missing in this interval, such as 18, do not have any neuropsychological test values in the ADNI database.) A sequence of length four uses the baseline, and months 6, 12, and 24 data; a sequence of length six also includes months 36 and 48, and a sequence of length eight includes months 60 and 72.

#### Neuropsychological test selections

Five neuropsychological test scores were used to assess the cognitive state of the subjects. The tests are MMSE, ADAS Q4, ADAS Cog-11, ADAS Cog-13, and FAQ. These tests have been used to demonstrate their effectiveness in the diagnosis and prediction of cognitive impairment [12]. Additionally, neuropsychological tests were used in a regression model [39] to determine the impact on change in CDR sum of scores, and “MMSE, FAQ, and ADAS-cog were identified as prognostic factors to detect cognitive decline in CDR-SB”. Other test scores, such as the Wechsler Logical Memory Delay (LDELTOTAL), are also available. However, the number of missing values of the latter test was greater than for the other tests, and the test was given less frequently (every 2 years as opposed to half-yearly or yearly). As a result, LDELTOTAL could not be used as a marker in this study.

### Training, validation and test data set creation

As noted earlier, the battery of neuropsychological tests is administered during scheduled visits that occur at the initial visit (baseline) and then at months 6, 12, 24, and beyond. All participants do not attend all of the scheduled visits, and even when they do, results for all scheduled tests are not available. Consequently, there are many missing values for all of the tests that are part of the ADNI study. In order to predict the cognitive status 2–4 years ahead, a minimum of four past test scores is needed for each test subject. As a result, sequences of lengths four, six, and eight were extracted for each of the five neuropsychological tests separately from the ADNI1, ADNI-GO, and ADNI2 data sets and combined to form the training and validation data for the test-score prediction LSTM models. No special care was taken to collect sequences for the same patient across the neuropsychological tests since the generic pattern of disease progression is what the LSTMs need to learn, not the progression pattern of the same patient across the tests.

Special care had to be taken to re-create or impute missing values. To use mostly real data values and reduce the number of imputations, it was ensured that subjects had no more than two missing test scores. Moreover, subjects who had a missing value on the last visit of a sequence of lengths four, six, or eight were also dropped. This constraint ensured that no model would learn the generic trend of disease progression using imputed values as the most recent data point. A simple average method was used to impute missing values. As an example, for a model of length four, a subject with missing data for month 12 was assigned the average of the baseline, month 6, and month 24 values. It is one of the many ways in which missing data can be imputed. This imputation method is advantageous since this method preserves the average of the sequence.

Imputed sequences of length six from the combined data set were used to generate the training and validation data for the LSTM models used to predict the fifth and sixth values. For example, for the LSTM model for predicting the fifth value, the model was trained with sequence values one through four as input data and sequence value five as the output data value. The model learned how the fifth value was associated with the previous four values using the training data and tested what it learned on the validation data to determine its accuracy. Similarly, for the sixth value prediction model, sequence values one through five were used as the input data, and value six was used as the output data value. Eighty percent of the rows were used to create the training set and the remaining 20% of rows made up the validation set. For the seventh and eighth element prediction models, a similar set of steps were followed using the combined data set containing sequences of length eight.

The MLPs need to learn to diagnose the cognitive state of a single patient given the predicted test score values of the five neuropsychological tests of that patient. Sequences of lengths four, six, and eight were extracted for a given RID for each of the five neuropsychological tests separately from the ADNI1, ADNI-GO, and ADNI2 datasets, yielding 15 different collections. The ADNI diagnosis values (CN, MCI, or AD) were also saved for each RID and for each sequence at the fourth, sixth, and eighth visits from the three data sets (ADNI1, ADNI-GO, and ADNI2). If the test score of any of the five tests at visits four, six, or eight and the corresponding ADNI diagnosis was not available, the RID was removed from the data set. Among the RIDs that remained, if any sequence for the five tests contained more than two unavailable scores, the RID was removed from the data set. The remaining test scores formed the core for generating the training and validation data for the diagnosis MLP models. A classification output value of 0, cognitively normal (CN), was generated for an ADNI diagnosis value of CN and a classification output value of 1, non-CN, was generated for ADNI diagnosis values of MCI and AD. For each sequence length, the data were then sorted according to the classification value (0 or 1), and 80% of the smaller of the two classified sets was combined with a matching number of sequences from the other set of sequences to create the training set. This procedure was carefully incorporated due to the models’ enhanced ability to learn if there are an equal number of 0s and 1s. Finally, the remaining sequences were pulled together to form the validation data set.

**Table 1:** Descriptive statistics of the 66 test dataset subjects

Age	#	Gender	#	Education	#	Race	#	Marital status	#
58–68	8	Female	29	8–11	3	Asian	2	Divorced	4
68–78	41	Male	37	12–15	22	Black	4	Married	49
78–88	17			16–20	41	White	60	Never married	1
								Widowed	12

To determine if a combination of neuropsychological tests can be used to predict the future cognitive state of an individual, it is necessary to create a test data set of individuals for whom longitudinal data of length eight (baseline to 72 months) are available for all of the five tests chosen for this study. After applying the same criterion of no more than two missing values, no missing diagnosis value, and ensuring that these sequences were not used as training or validation data for any LSTM or MLP model, only 66 patients who had values for all the five neurological tests could be obtained from the oldest cohort—ADNI1. Descriptive statistics for this cohort are given in Table 1.

**Table 2:** Test score cutoffs and normalizations

Test	Normal cutoff	Min	Max	Normalization
MMSE	≥ 28	0	30	Value/30
ADAS4	≤ 5	0	10	Value/10
ADAS11	≤ 10	0	70	Value/70
ADAS13	≤ 13	0	85	Value/85
FAQ	≤ 2	0	30	Value/30

Table 2 provides the range of test score values for each test, along with their cutoff scores for normal values which were obtained from the different references discussed in Sects. 1 and 2. The last column of the table provides the normalization calculations used for each test to convert the values to a scale of 0 to 1. Even though the cutoff values were not used to determine the classification output values of the MLP models, these cutoff values were used to determine the efficacy of the sequence prediction LSTM models and to determine the contribution of the normal and abnormal patients towards the overall diagnosis as discussed in a later section.

#### Model creation and application

Each diagnosis and sequence prediction model was trained using its training data and validated using its validation data. The loss curve was plotted for each model, and the model and the loss curve were saved. The models with the highest prediction accuracy were saved for later use in the test data prediction phase.

**Table 3:** Number of observations for training and validation of sequence prediction RNNs

Test	Training 36th and	Training 60th and	Validation 36th and	Validation 60th and
	48th months	72nd months	48th months	72nd months
MMSE	458	230	115	57
ADAS4	458	220	115	55
ADAS11	441	202	110	50
ADAS13	437	209	109	52
FAQ	466	250	116	63

**Table 4:** Number of observations for training and validation of diagnosis MLPs

Diagnosis time	Training	Validation
Month 24	632	156
Month 48	428	156
Month 72	264	104

Table 3 gives the number of unique RIDs used to train and validate the sequence prediction models. Table 4 gives the number of unique RIDs used to train and validate the fourth step, sixth step, and eighth step diagnosis models. The numbers show that for each test, the number of observations for the data sets decreases when going from sequences of length six to sequences of length eight since the number of RIDs that have missing values over a 72-month period is much higher than it is over a 48-month period.

**Fig. 1:**
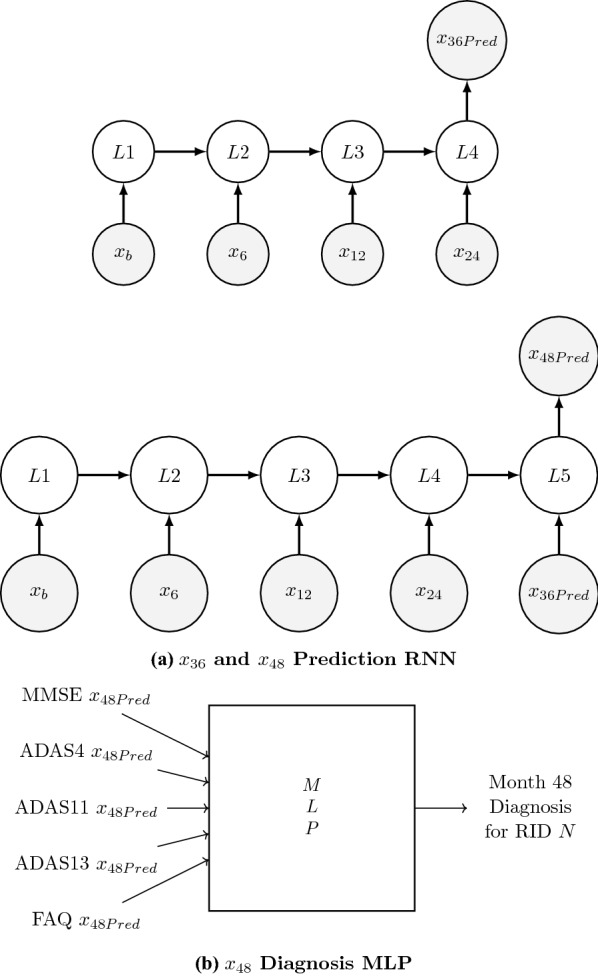
48-month sequence prediction for a neuropsychological test for RID *N*

**Fig. 2:**
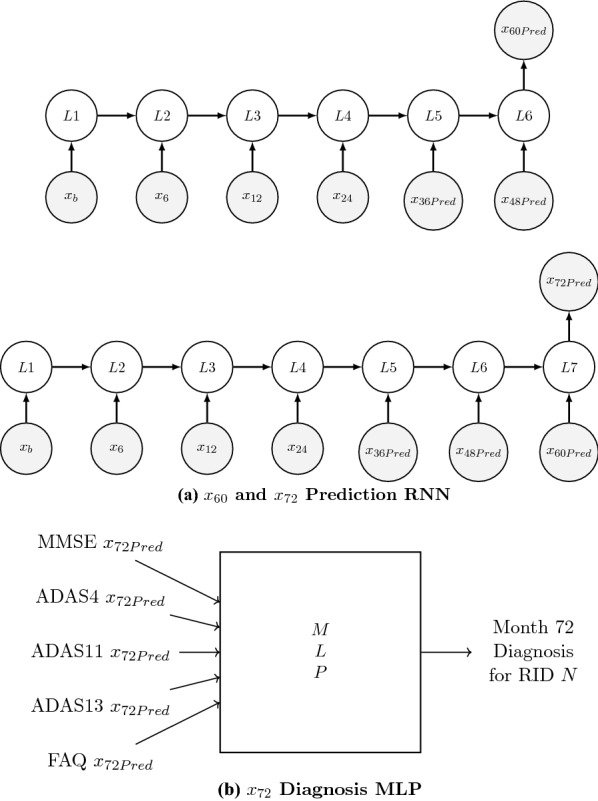
72-month sequence prediction for a neuropsychological test for RID *N*

Figures 1 and 2 show the schematic of how the different neural network models were used. The main goal of this study lies in the combination of the predictive and diagnostic models. The combination of the models is used on the final test data created for each neuropsychological test. The final test data set is the set of RIDs that is used to test the accuracy and practicality of the study, and it consists of 66 patients who all have test scores for each of the neuropsychological tests. As shown in Fig. 1a, the first four values of a sequence for a specific test and for a specific RID, N, say, were fed into the best fifth-element sequence prediction model to predict the month 36 value for the test ($x_{36\text{Pred}}$). Then, the same four values along with $x_{36\text{Pred}}$ were fed into the best sixth-element sequence prediction model to predict the month 48 value for the test ($x_{48\text{Pred}}$). This process was repeated for every test for RID N to obtain the $x_{48\text{Pred}}$ values for all tests. Then, as shown in Fig. 1b, all of the $x_{48\text{Pred}}$ values were fed to the best month-48 diagnosis model to diagnose the CN–non-CN status of RID N. Figure 2 shows the same process repeated for the generation of ($x_{72\text{Pred}}$) for all the tests and the corresponding diagnosis of the CN–non-CN status for RID N. Then these diagnosis predictions, for months 48 and 72, were compared to the real diagnosis given by ADNI for RID N (using DX values) at months 48 and 72, respectively, to determine the accuracy of the combined model.

**Fig. 3:**
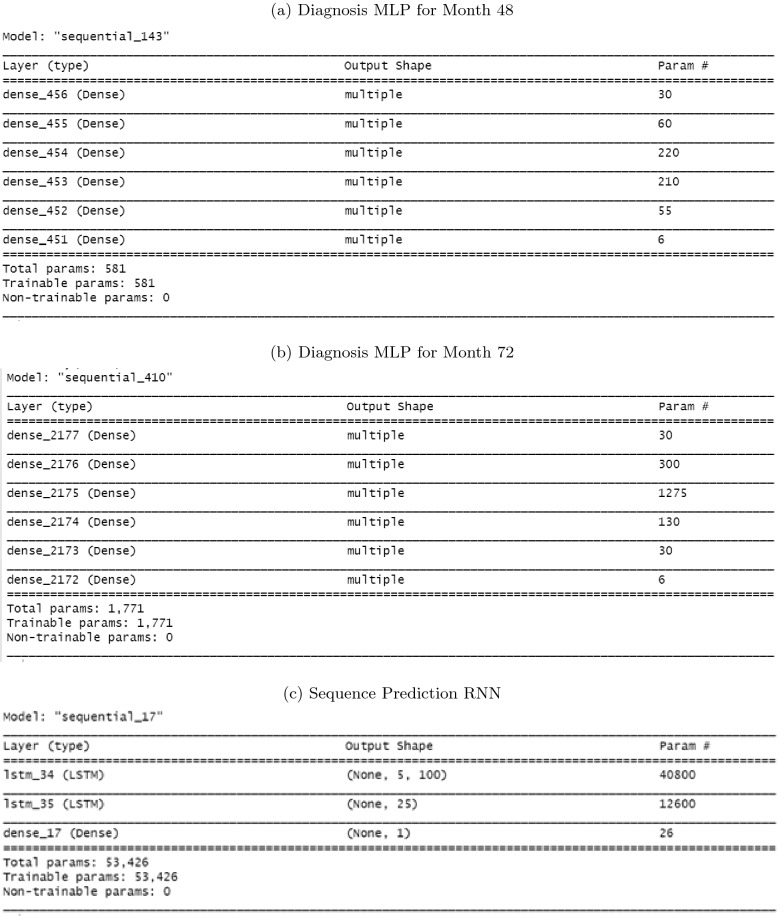
Summary of diagnosis MLPs and sequence prediction RNN models

Figure 3 summarizes the parametric structure of the three types of neural network models that were used in this study. Figure 3a shows the structure of the diagnosis MLP that was used to diagnose the five neurological test scores at month 48. The neural network has an input layer, a “sigmoid” output later, and four hidden layers. Figure 3b shows the structure of the diagnosis MLP that was used to diagnose the five neurological test scores at month 72. It also has an input layer, a “sigmoid” output later, and four hidden layers but uses a different number of neurons in each hidden layer. The ‘relu’ activation was used for every non-output layer and the “mean-squared-error” loss function and the ‘adam’ optimizer were used to compile the models. Figure 3c shows the common structure of the sequence prediction LSTMs that were used to predict the five neurological test scores. Two layers of LSTMs were used followed by a dense output layer. The ‘relu’ activation was used for every layer and the “mean-squared-error” loss function and the ‘adam’ optimizer were used to compile the models.

## Results

There are three sets of results for this work, and they relate to (1) accuracies of the diagnosis of cognitive states, (2) how closely the predicted values of the test scores follow the trend of the actual values in the validation data set 2 years (sixth value) and 4 years (eighth value) after month 24, and (3) accuracies of the diagnosis when the sequence prediction LSTMs and the diagnosis MLPs are combined to predict on the test data set.

### Diagnosis models’ accuracies

The diagnosis models learn from the training data to determine which combination of the five test scores should be assigned a CN (0) value and which combination should be assigned a non-CN (1) value. When the trained models are applied to the validation data, the models’ assignments of 0 and 1 are compared with the labeling of 0 (ADNI diagnosis CN, DX = 1) and 1 (ADNI diagnosis MCI/AD, DX = 2/3) of the validation data sequences to arrive at the accuracy results.

**Table 5:** Diagnosis models’ accuracies

Diagnosis time	Accuracy (%)
Month 24	85.25
Month 48	78.85
Month 72	77.88

Table 5 gives the accuracies of the diagnosis models. The diagnosis at month 48 is 78.85% accurate and the diagnosis at month 72 is 77.88% accurate. The accuracy values were determined by taking the proportion of correct diagnoses to the total number of diagnoses.

**Fig. 4:**
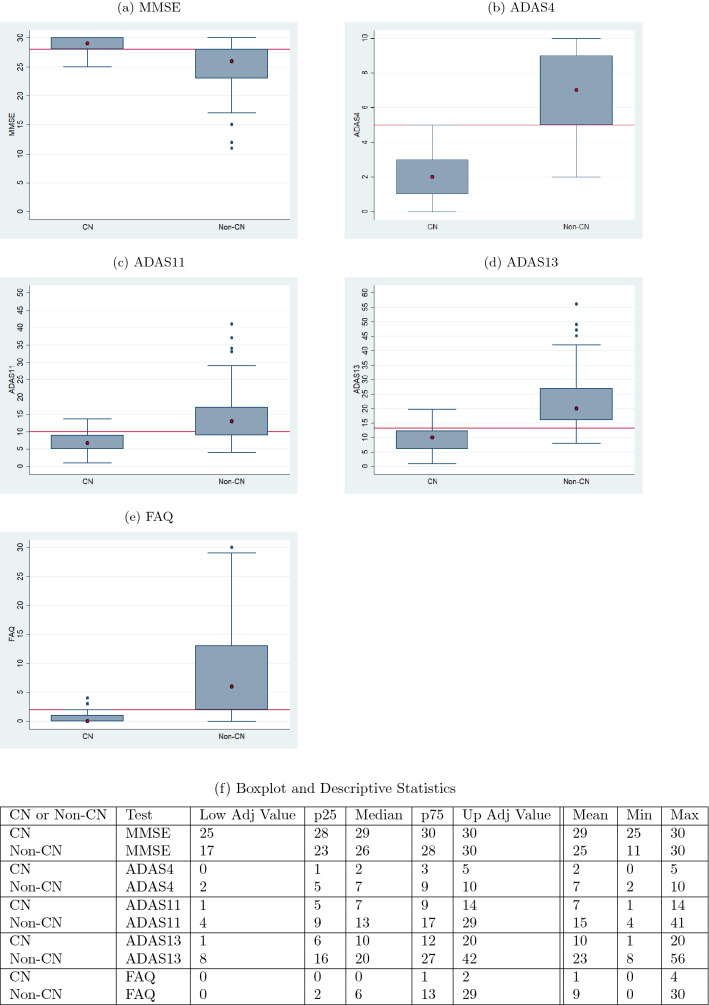
Ranges of validation data split by the predicted diagnosis (CN or non-CN) assigned by the diagnosis model at month 48 (with normal cutoffs)

**Fig. 5:**
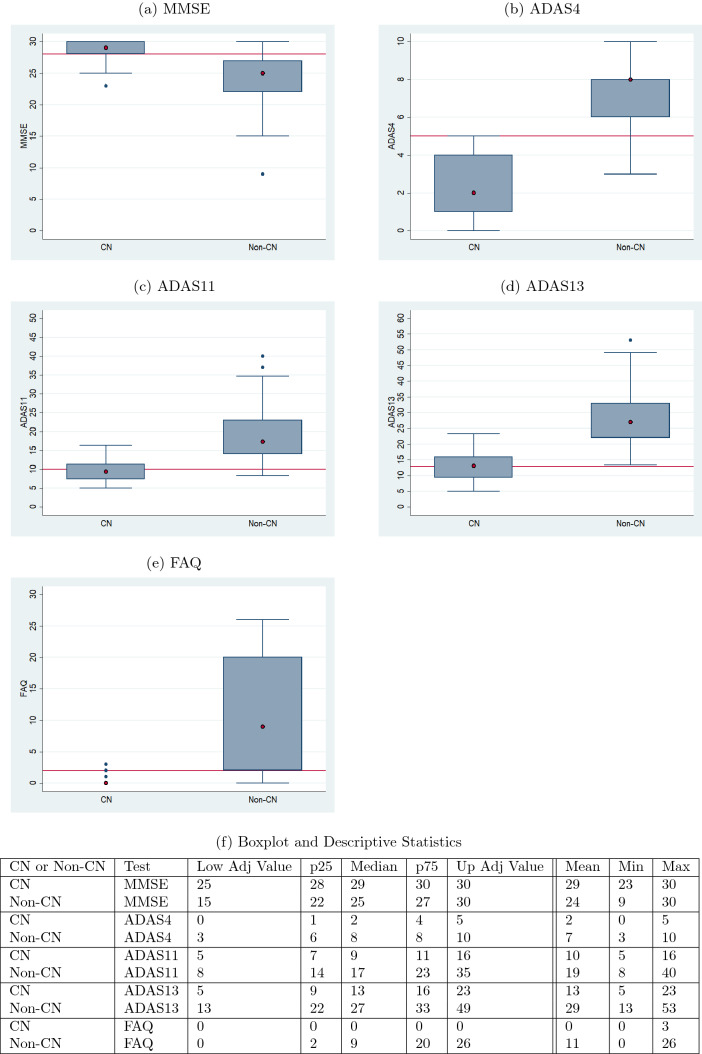
Ranges of validation data split by the predicted diagnosis (CN or non-CN) assigned by the diagnosis model at month 72 (with normal cutoffs)

The boxplots of Figs. 4 and 5 serve as a corroboration of the accuracies noted above. The plots also corroborate the accuracy of the normal–abnormal cutoff values shown in Table 2. The boxplots for each graph are split into two groups, CN and non-CN. CN depicts the range of scores diagnosed as 0 by the diagnosis models, and non-CN depicts those diagnosed as 1. The stark contrast in the ranges of the CN and non-CN boxplots shows that the diagnosis models were able to effectively learn for all of the five tests. The tables in Figs. 4f and 5f contain the descriptive statistics of the boxplots along with the minimum value, maximum value, and the mean of the boxplot ranges for each of the five tests.

**Fig. 6:**
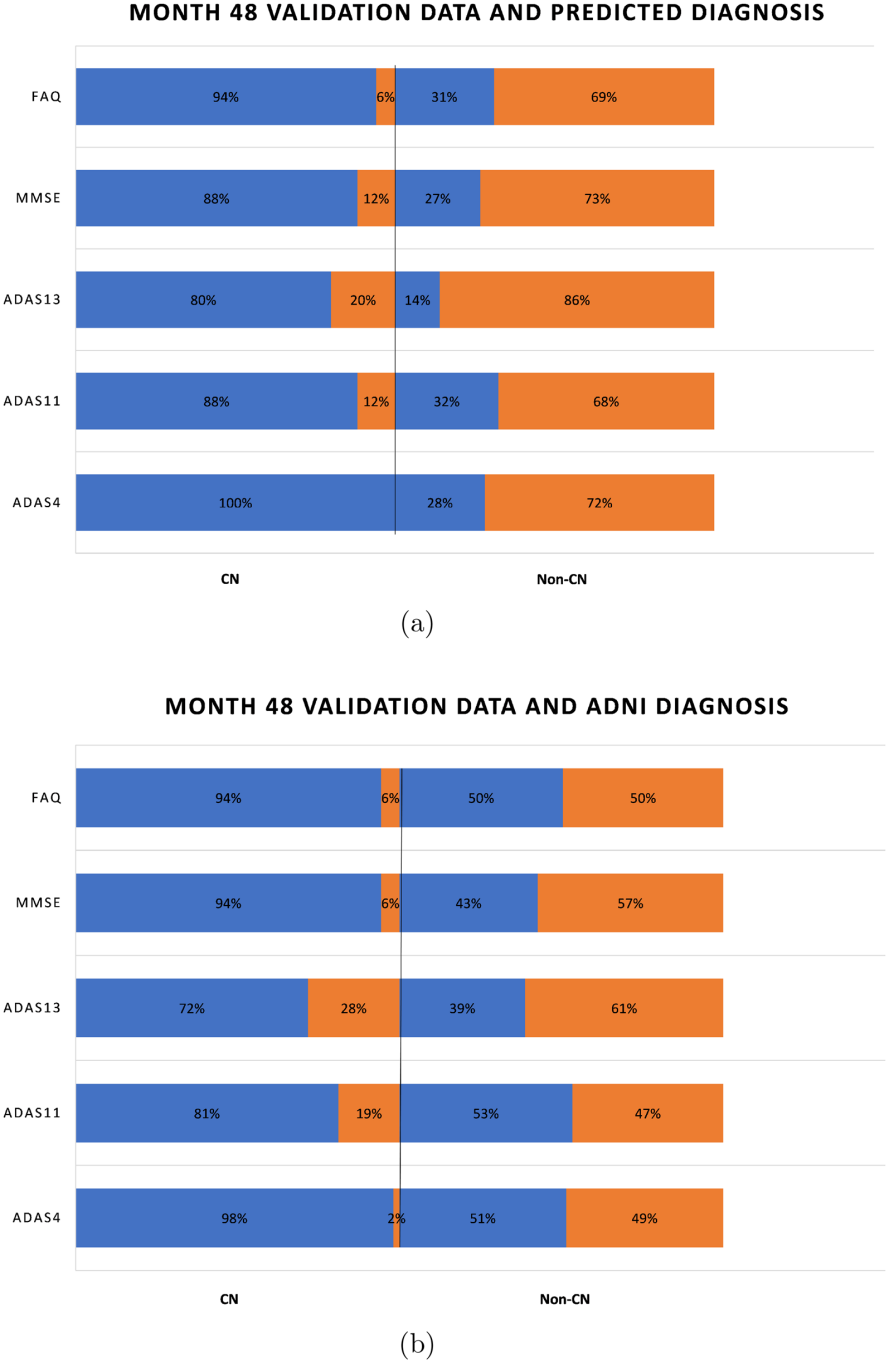
Proportion of correct and incorrect diagnosis in CN and non-CN categories for validation data at month 48

An aspect of the boxplots of Fig. 4 that deserves special mention is that even though the prediction accuracies are in the high seventies, there are still some RIDs that are incorrectly classified. For example, for MMSE (Fig. 4a), the CN group contains some RIDs that are in the abnormal range ($< 28$) and the non-CN group contains some RIDs that are in the normal range (≥ 28). Figure 6a shows a bar graph for all the five tests in which the CN and non-CN groups are further split into normal and abnormal percentage values based on the cutoff values for the test from Table 2. For example, for MMSE, the CN group contains 88% RIDs that are in the normal range and 12% RIDs that are in the abnormal range. It is these 12% abnormal RIDs that run the risk of being misdiagnosed as normal and hence dropped from further monitoring and treatment. Similarly, for MMSE, the non-CN group contains 31% RIDs that are in the normal range and 69% RIDs that are in the abnormal range. Once again, it is the 31% normal RIDs that run the risk of being monitored and treated causing a waste of valuable resources. Such misdiagnosis is inevitable in the context of machine learning approaches, especially when training data are scarce. But one important observation that should be made in regard to the diagnosis reached by ADNI, as shown in Fig. 6b, is that the mix of normal−abnormal misdiagnosis percentages are not any better when we consider the validation data and group them by the ADNI DX value (CN and non-CN, that is, MCI/AD). As seen in Fig. 6b, for MMSE, say, the non-CN group contains 43% RIDs that are in the normal range (compared to 27% using the predicted diagnosis of our models) and run the risk of being monitored and treated causing a waste of valuable resources. All the tests in Fig. 6a, when compared with the corresponding tests in Fig. 6b show that our prediction and diagnosis models perform better in reducing the misdiagnosis percentage values thereby promising to be a cost-effective solution for the initial screening of Alzheimer’s patients.

**Fig. 7:**
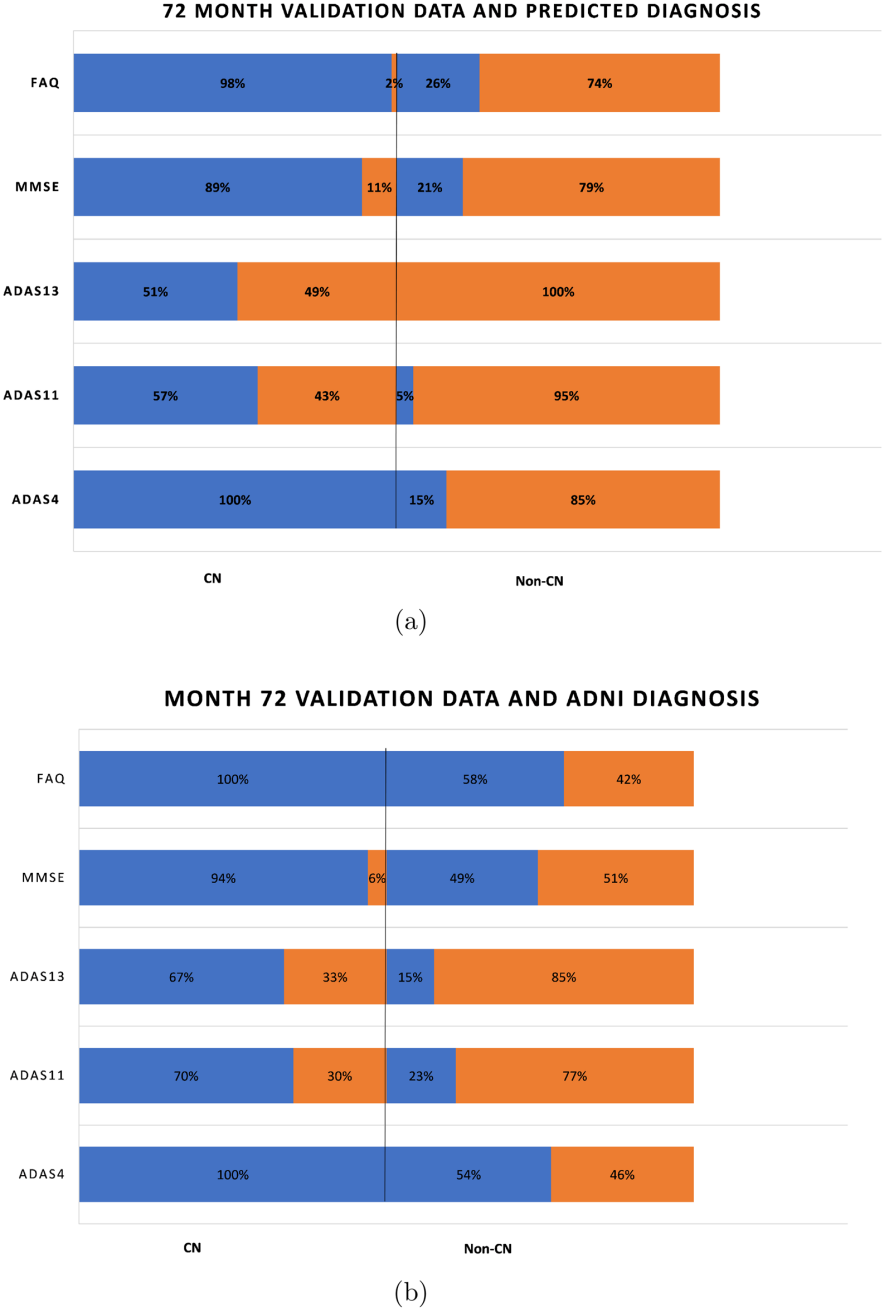
Proportion of correct and incorrect diagnosis in CN and non-CN categories for validation data at month 72

Figure 7a and b similarly capture the misdiagnosis percentage values for predicted diagnosis and ADNI diagnosis outcomes at month 72 and it is clear once again that our prediction and diagnosis models perform better in reducing the misdiagnosis percentage values.

### Performance of the sequence prediction models

**Fig. 8:**
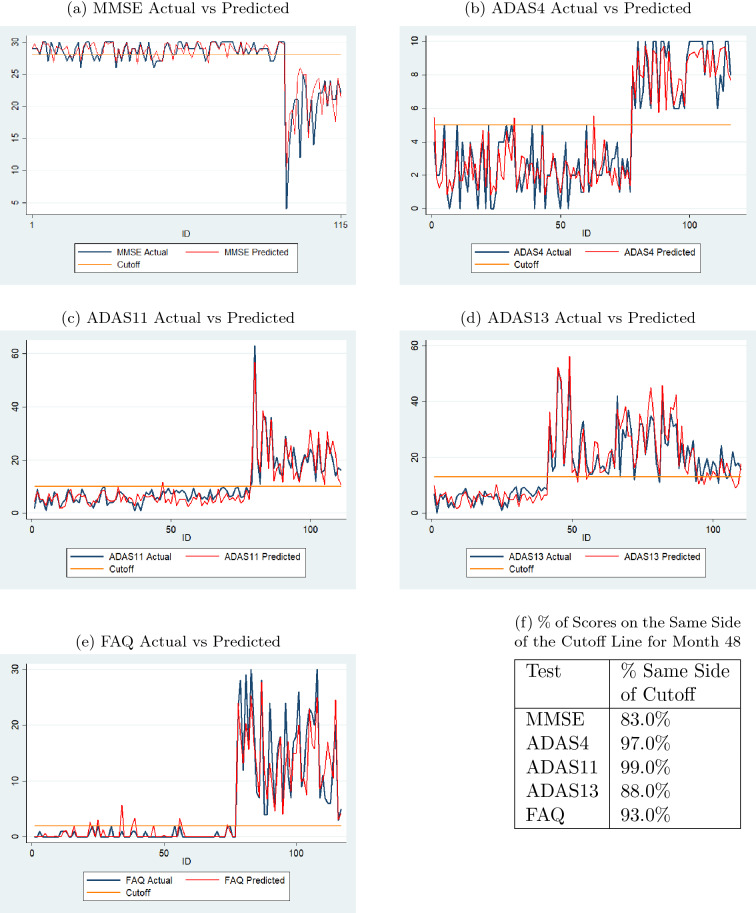
Comparison of actual and predicted values by sequence prediction models at month 48 for validation data (with normal cutoffs)

Figure 8 consists of a series of graphs that demonstrate the performance of the sequence prediction models when predicting the sixth value based on the first four actual values and the predicted fifth value. The validation data sets were chosen for these figures. The blue line depicts the actual sixth value of each of the subjects, while the red line depicts the predicted sixth value using the best sequence prediction model. The horizontal yellow line represents the threshold that differentiates test scores defined as CN or non-CN from Table 2. The comparisons show that even if the predicted values are not exactly identical to the actual values, they are close and more importantly, follow the general trend of the actual data. If the predicted values did not follow the general trend, the diagnoses based on these values would not be accurate. Moreover, the inaccuracy is of greater concern if the predicted and actual values were on two different sides of the cutoff line. The percent values in the table in Fig. 8f show that the percentages of predicted scores on the same side of the cutoff lines are high for all tests for month 48.

**Fig. 9:**
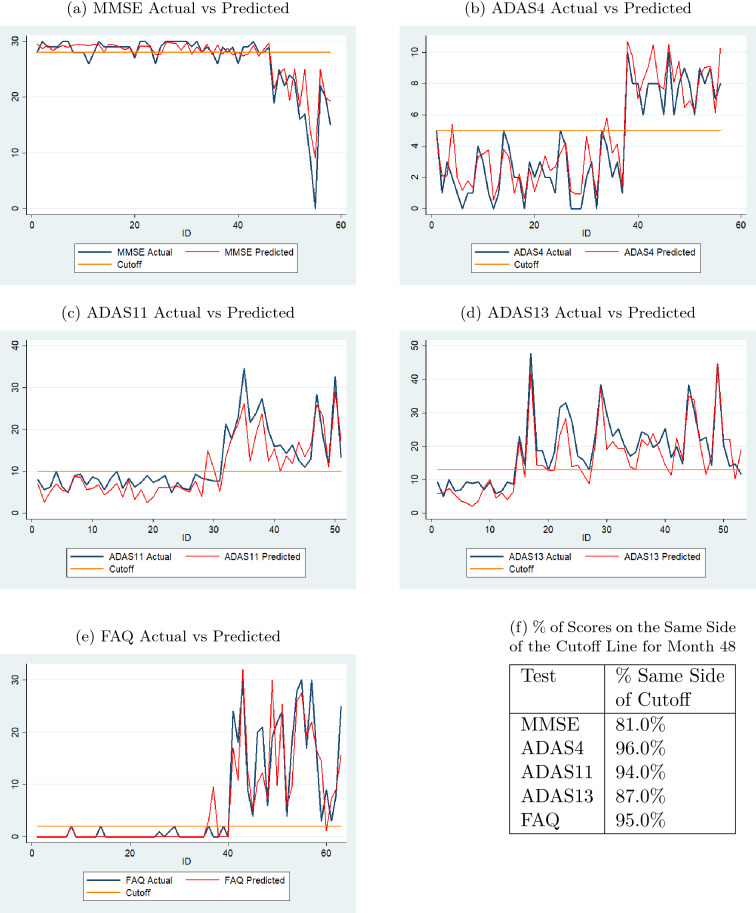
Comparison of actual and predicted values by sequence prediction models at month 72 for validation data (with normal cutoffs)

Figure 9 displays the performance of the sequence prediction models when predicting the eighth value, the 72nd month, based on the first four actual values and the predicted fifth, sixth, and seventh values. In this case, as well, the data are for the individuals in the validation data sets. The results are similar to those reported above for predicting the sixth value. The percent values in the table in Fig. 9f show that the percentages of predicted scores on the same side of the cutoff lines are high for all tests for month 72.

**Fig. 10:**
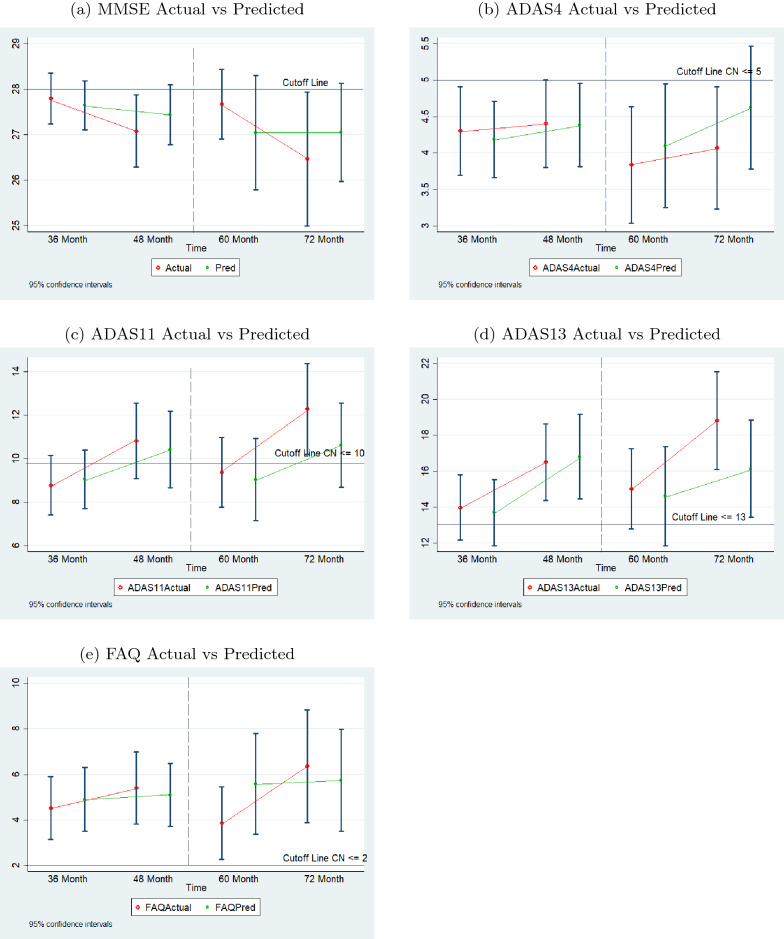
95% confidence intervals of actual and predicted values for sequence prediction models for validation data (with normal cutoffs)

Figure 10 shows the 95 percent confidence intervals of actual and predicted values for validation data for each test. Since the validation data sets for predicting the month 36 and month 48 values are different from that for predicting the month 60 and month 72 values, the means of the 48-month and 60-month intervals could not be connected and the two sections of each chart are separated by a dashed line. The charts demonstrate that the predicted values follow the general trend of the actual data for each test and the spread of the predicted values are comparable to that of the actual data.

**Fig. 11:**
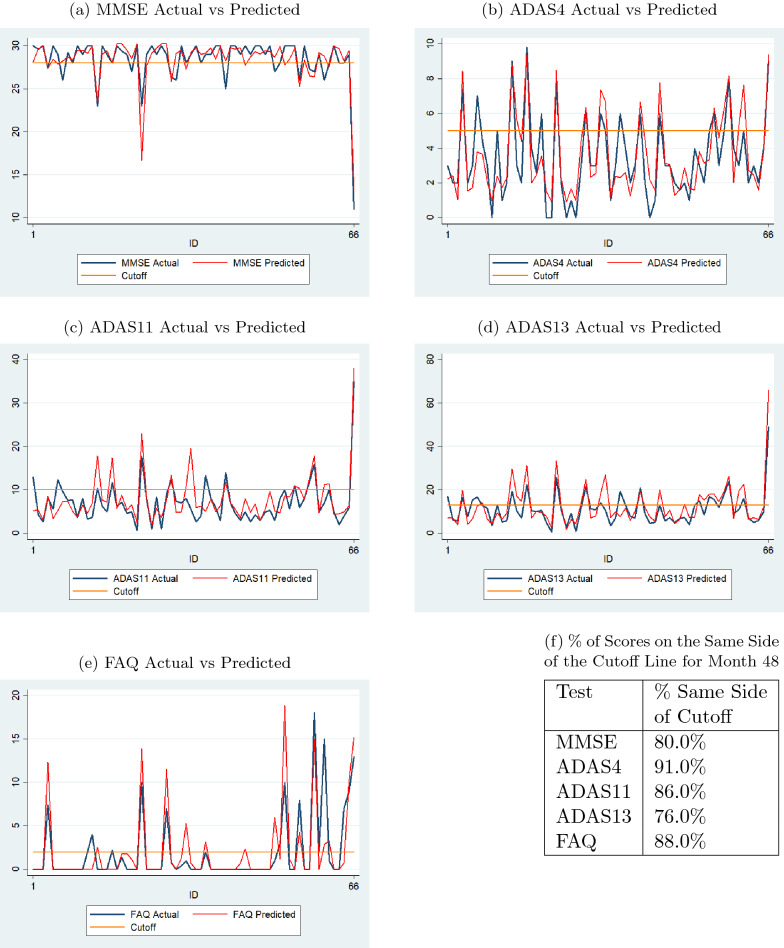
Comparison of actual and predicted values by sequence prediction models at month 48 for 66 test dataset subjects (with normal cutoffs)

**Fig. 12:**
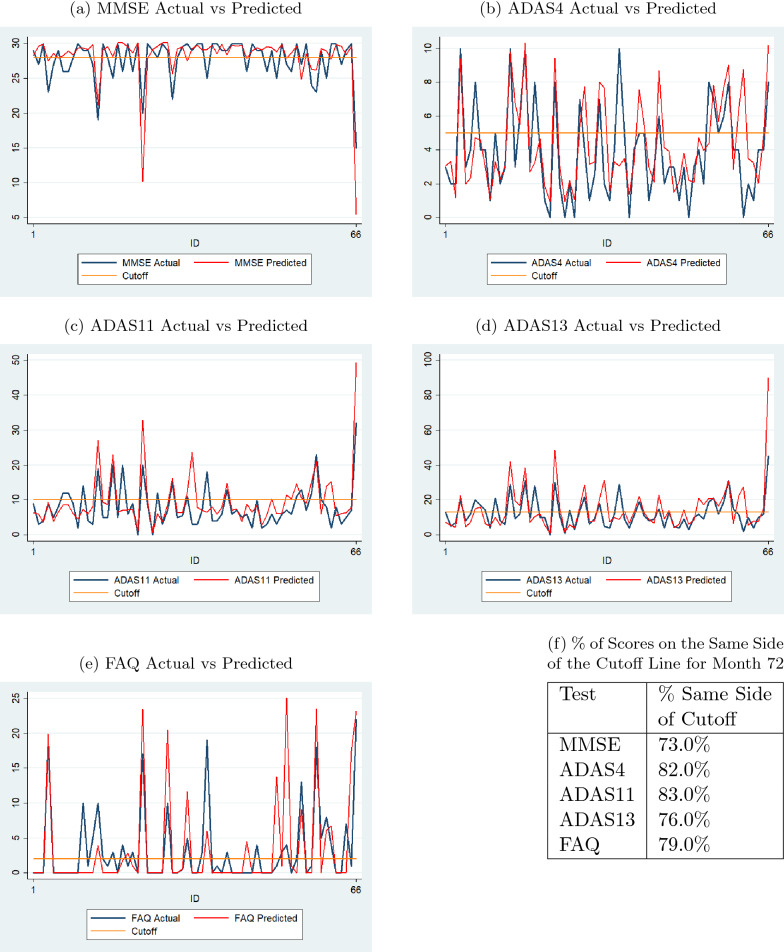
Comparison of actual and predicted values by sequence prediction models at month 72 for 66 test dataset subjects (with normal cutoffs)

Figures 11 and 12 display results, similar to those in Figs. 8 and 9, for the 66 individuals in the test data set. The percent values in the table in Fig. 11f show that the percentages of predicted scores on the same side of the cutoff lines are high for all tests for month 48. The same is true for month 72 as shown in the table in Fig. 12f.

**Fig. 13:**
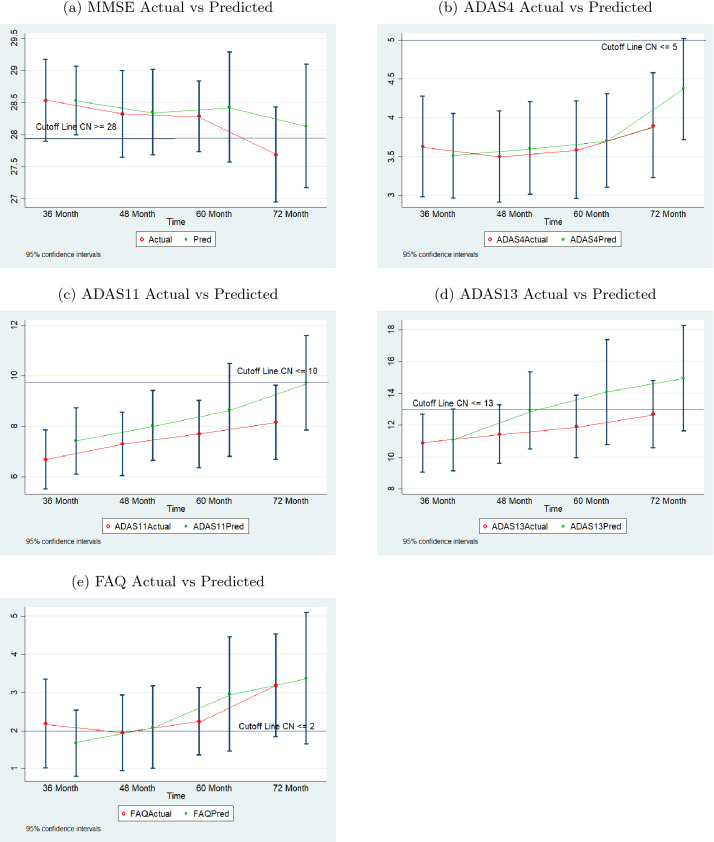
95% confidence intervals of actual and predicted values for sequence prediction models for test data (with normal cutoffs)

Figure 13 shows the 95 percent confidence intervals of actual and predicted values for test data for each test. The charts demonstrate that the predicted values follow the general trend of the actual data for each test and the spread of the predicted values are comparable to that of the actual data.

### Combined model’s accuracy

The key objective of this study is to determine if neuropsychological test scores can be used to predict if an individual will remain cognitively normal in the next 2–4 years. If the models, when combined together, predict that a patient will not remain CN over the next 2–4 years, then those individuals should be monitored more frequently and are likely to be recommended to undergo further testing.

Tables 6 and 7 collectively show that the combined model, when applied to the test data set, is successful in predicting that an individual will be cognitively normal at the end of the next 2 years with 84.62% accuracy. This accuracy drops to 83.33% for predictions that are 4 years in the future. Similarly, the combined model is successful in predicting that an individual will be cognitively abnormal at the end of the next 2 years with 78.9% accuracy. This accuracy rises to 82.8% for predictions that are 4 years in the future. Table 8 shows the overall accuracies of the combined model at months 48 (82.76%) and 72 (83.08%) which reiterates the efficacy of this this novel, two-steptechnique. The subjects predicted to be cognitively abnormal are good candidates for more invasive testing and are likely to serve as good candidates for clinical trials for AD treatments. Hence, even though the ADNI diagnosis is not solely based on these five neuropsychological tests, our technique shows that if we use just the outcomes of these five tests, we can determine with a high degree of accuracy how the disease will progress for patients for whom there are no other data available to aid the diagnosis.

**Table 6:** Combined model’s prediction accuracy by category at month 48

Combined model’s predictions		ADNI’s assessment			Accuracy (%)
Diagnosis	No. of subjects	CN	Non-CN	NA	
CN	44	33	6	5	84.62
Non-CN	22	4	15	3	78.9

**Table 7:** Combined model’s prediction accuracy by category at month 72

Combined model’s predictions		ADNI’s assessment			Accuracy (%)
Diagnosis	No. of subjects	CN	Non-CN	NA	
CN	37	30	6	1	83.33
Non-CN	29	5	24	0	82.8

**Table 8:** Combined model’s overall diagnosis accuracies

Diagnosis time	Accuracy (%)
Month 48	82.76
Month 72	83.08

**Fig. 14:**
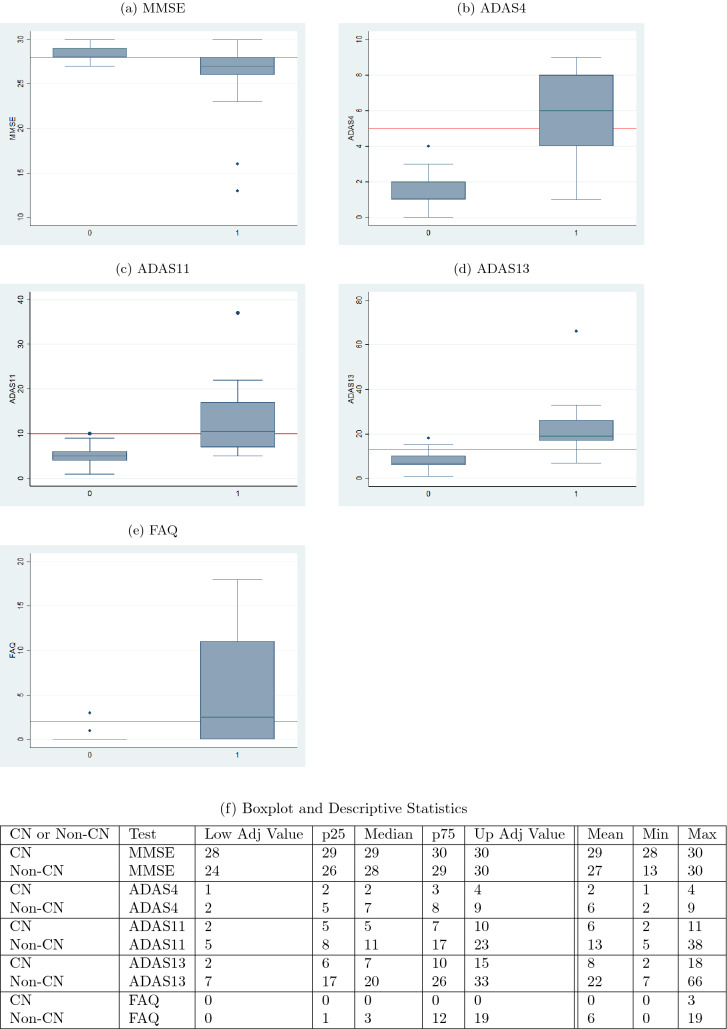
Ranges of test data split by the predicted diagnosis (CN or non-CN) assigned by the diagnosis model at month 48 (with normal cutoffs)

The boxplots of Figs. 14 and 16 serve as a corroboration of the accuracies noted above. The plots also corroborate the accuracy of the normal–abnormal cutoff values shown in Table 2. The boxplots for each graph are split into two groups, CN and non-CN. CN depicts the range of scores diagnosed as 0 by the diagnosis models, and non-CN depicts those diagnosed as 1. The stark contrast in the ranges of the CN and non-CN boxplots shows that the diagnosis models were able to predict very effectively on data that it had not seen before during training and validation. The tables in Figs. 14f and 16f contain the descriptive statistics of the boxplots along with the minimum value, maximum value, and the mean of the boxplot ranges for each of the five tests.

**Fig. 15:**
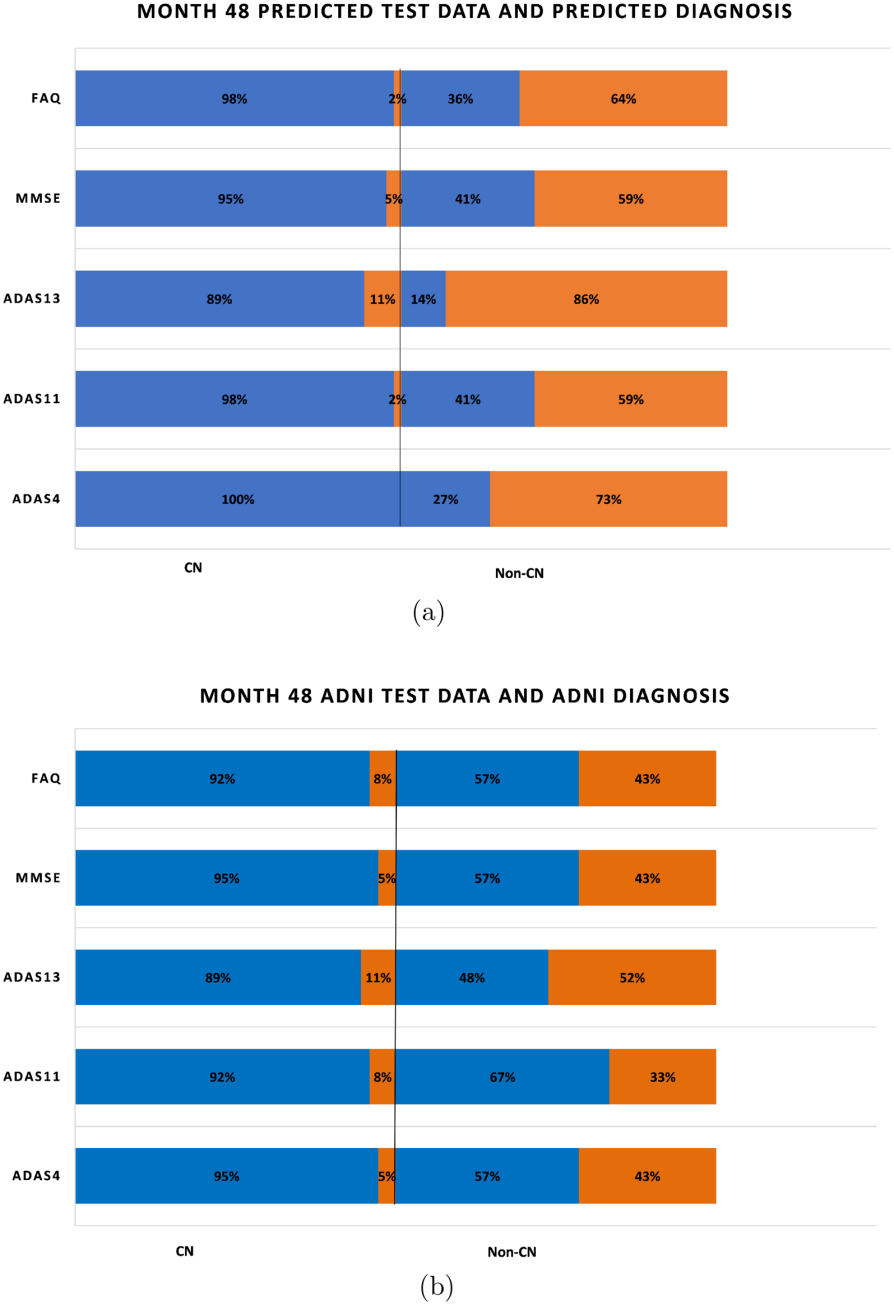
Proportion of correct and incorrect diagnosis in CN and non-CN categories for test data and predicted test data at month 48

**Fig. 16:**
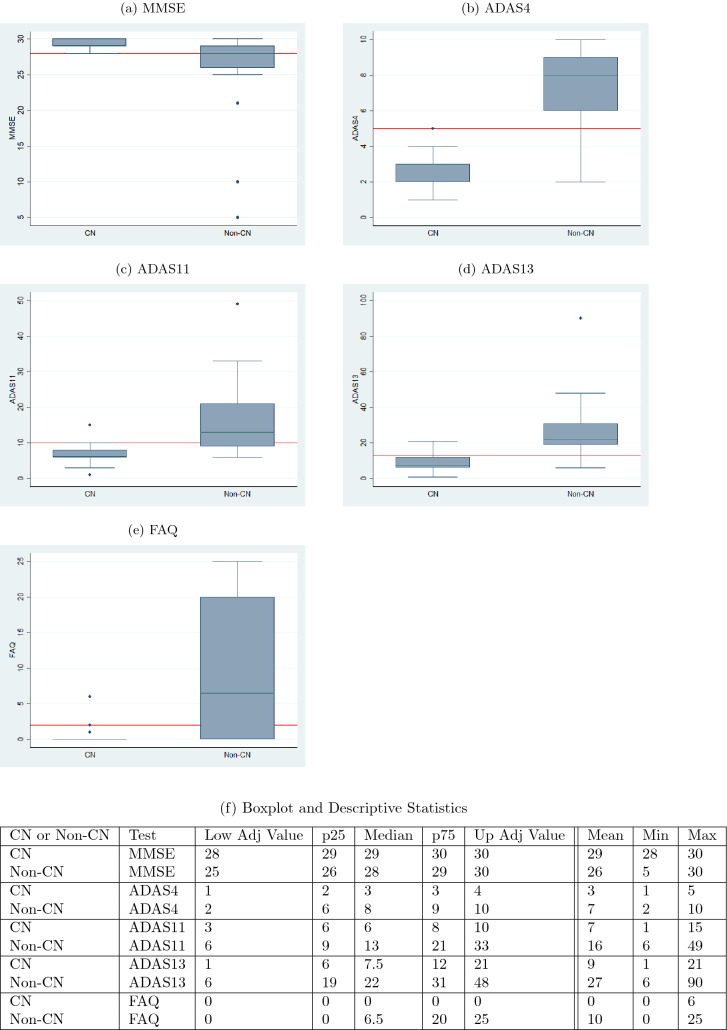
Ranges of test data split by the predicted diagnosis (CN or non-CN) assigned by the diagnosis model at month 72 (with normal cutoffs)

**Fig. 17:**
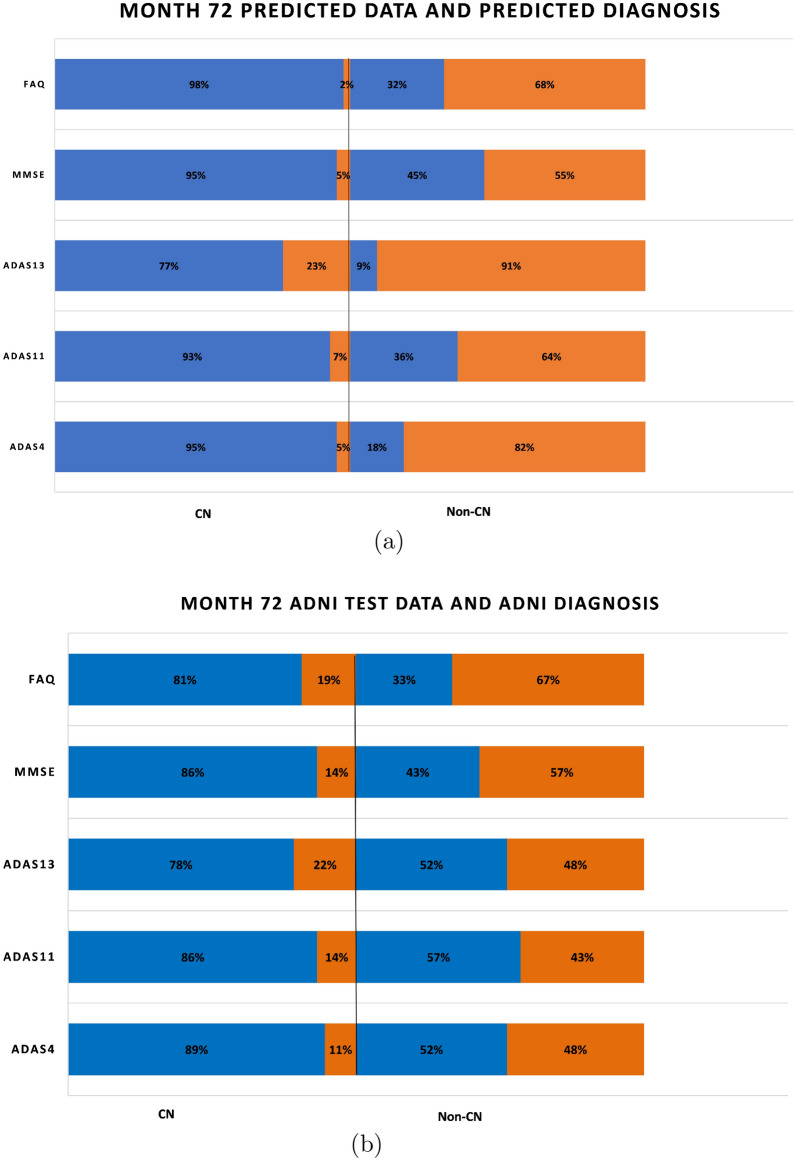
Proportion of correct and incorrect diagnosis in CN and non-CN categories for test data and predicted test data at month 72

Figure 15a shows a bar graph for all the five tests in which the CN and non-CN groups are further split into normal and abnormal percentage values based on the cutoff values for the test from Table 2. For example, for MMSE, the CN group contains 98% RIDs that are in the normal range and 2% RIDs that are in the abnormal range. Similarly, for MMSE, the non-CN group contains 41% RIDs that are in the normal range and 59% RIDs that are in the abnormal range. As before, in regard to the diagnosis reached by ADNI, as shown in Fig. 15b, the mix of normal–abnormal misdiagnosis percentages is not any better when we consider the test data and group them by the ADNI DX value (CN and non-CN, that is, MCI/AD). As seen in Fig. 15b, for MMSE, say, the non-CN group contains 57% RIDs that are in the normal range (compared to 41% using the predicted diagnosis of our models) and run the risk of being monitored and treated causing a waste of valuable resources. All the tests in Fig. 15a, when compared with the corresponding tests in Fig. 15b show that our prediction and diagnosis models perform better in reducing the misdiagnosis percentage values thereby promising to be a cost-effective solution for the initial screening of Alzheimer’s patients.

Figures 16 and 17 similarly capture the misdiagnosis percentage values for predicted diagnosis and ADNI diagnosis outcomes at month 72 and it is clear once again that our prediction and diagnosis models perform better in reducing the misdiagnosis percentage values.

## Conclusion

The contribution of this work is the demonstration that simple neuropsychological tests can be used on their own to predict the future cognitive state of individuals. Using data from hundreds of subjects who participated in the ADNI project, this study used LSTM models to predict future values of their tests and used MLPs to diagnose individuals as CN or non-CN at a future date. For a cohort of 66 test subjects, the test scores of all five tests were combined to generate a prediction of their future cognitive states. These predictions were then compared with the actual CN or MCI/AD status assigned by ADNI. The results show that individuals who are predicted by the model to continue to remain CN in all of the tests are highly likely to remain cognitively normal in real life over the next 2 to 4 years. Individuals who are predicted to have test scores outside of normal ranges are likely to experience cognitive impairment in the future. These individuals should be monitored and treated.

The goal of this work was not to supersede the cognitive assessments made by ADNI. Instead, the goal was to determine whether the cognitive assessments made by ADNI could be used as outputs to train MLPs which relied only on a subset of the set of features that ADNI gathers for each participant (namely, the five neuropsychological tests). Data for these five features are relatively easy to collect and the two-step neural network algorithm can be used to forecast cognitive status for a 2- to 4-year window.

The analysis of this paper focused on only five tests for which data over at least a 72-month period was available in the ADNI database for a large number of individuals and there were no more than two missing values for each participant. The combined model’s structure allows one to introduce any test for which sufficient longitudinal data are available from ADNI or any other data source such as NACC (National Alzheimer’s Coordinating Center).

The benefit of developing a tool based on neuropsychological test scores that can predict with high accuracy the likelihood of an individual experiencing cognitive difficulties in the next few years derives from the low cost of administering these tests. If these tests become routine for individuals above a certain age or who have some risk factors for developing cognitive impairment, longitudinal performance data can be used with a high level of accuracy to determine which patients will require close monitoring. As treatments for Alzheimer’s disease continue to develop, this ability to determine who will require close monitoring can allow more invasive and expensive tests to be reserved for them. This can also allow for early intervention, which can be crucial in the treatment or prevention of the disease.

## Data Availability

Data used in this project are available from the Alzheimer’s Disease Neuroimaging Initiative (ADNI). Users need access permission from ADNI.
